# Diversification of DIX domain-containing proteins in the SAR supergroup

**DOI:** 10.1128/mbio.03966-24

**Published:** 2025-04-16

**Authors:** Maria-Myrto Kostareli, Timo Westerink, Gabriel Couillaud, Maaria Peippo, Francine Govers, Dolf Weijers, Edouard Evangelisti

**Affiliations:** 1 Institut Sophia Agrobiotech, UMR INRAE 1355, CNRS 7254, Université Côte d’Azur 131973, Sophia Antipolis, Provence-Alpes-Côte d'Azur, France; 2 Laboratory of Phytopathology, Wageningen University 593528 , Wageningen, Gelderland, the Netherlands; 3 Laboratory of Biochemistry, Wageningen University 593528 , Wageningen, Gelderland, the Netherlands; The University of British Columbia, Vancouver, British Columbia, Canada

**Keywords:** motility, stramenopiles, DIX, bigram

## Abstract

Polarity establishment is crucial for development, cellular organization, and signaling in living organisms. In animals and plants, this process involves DIX domain-containing proteins (DDPs) that assemble into oligomers via head-to-tail DIX polymerization, facilitating localized protein aggregation. This study uncovers the unexpected diversity of DDPs within the SAR supergroup, characterizing four DDPs with novel domain combinations conserved in Stramenopiles and Alveolates. These proteins are predominantly found in micro-swimmers and species with a motile stage in their life cycle. We hypothesize that DDPs from these eukaryotic lineages may be involved in cell polarity-related processes, including those associated with motility. Our work provides insights for further investigations of DDPs in protists and will enable the development of evolution-informed control strategies against pathogens and parasites within this clade.

## OPINION/HYPOTHESIS

Polarity establishment is a fundamental process guiding development, cellular organization, and signaling in living organisms. For instance, the body axis of animal embryos is determined by the Wnt/β-catenin signaling pathway, a central mechanism for embryonic development (1). This pathway relies on the assembly of signaling complexes termed signalosomes to prevent β-catenin degradation, enabling downstream regulation of target genes (2). Wnt signalosomes involve the oligomerization of DISHEVELLED (DVL) and AXIN. These proteins contain a conserved 100-residue region with a ubiquitin-like fold, known as the DIX domain (3), which reversibly assembles by head-to-tail polymerization (4). Recently, DIX domain-containing proteins (DDPs) have been characterized in plants (5). These proteins, called SOSEKIs, associate with the plasma membrane at specific corners of plant cells, acting as a molecular compass that drives cell polarity (5, 6). Building on these findings, we hypothesize that DDPs may have been recruited for similar processes in other eukaryotic lineages.

### The genomes of stramenopile and alveolate species encode novel DIX domain-containing proteins

**Fig 1:**
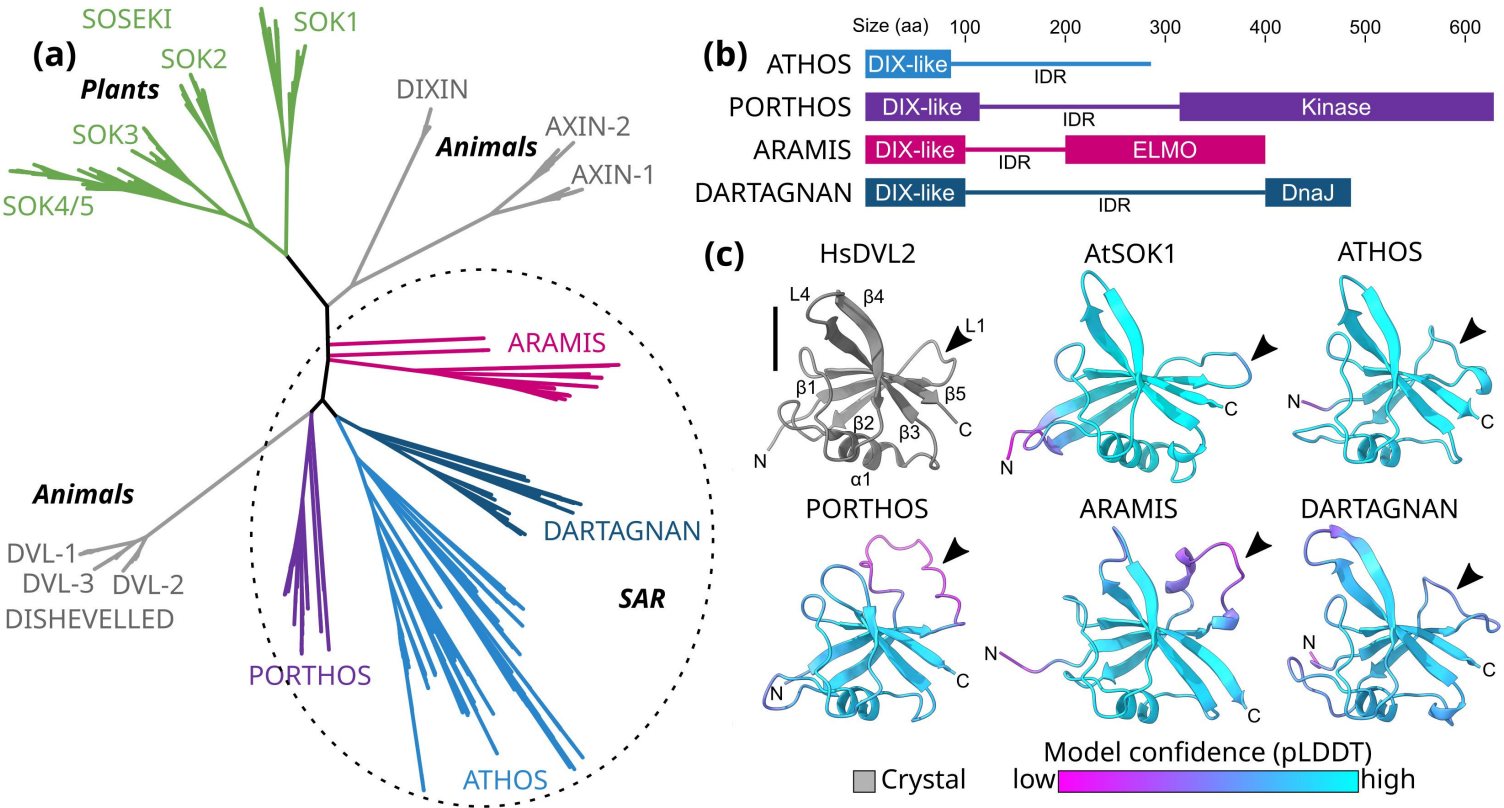
DIX domain-containing proteins diversify in the SAR supergroup. (**a**) Phylogenetic relationships of DIX domain-containing proteins (DDPs) in eukaryotes. Subclades corresponding to plant (SOSEKI) and animal (DISHEVELLED, AXIN, and DIXIN) DDPs are shown in green and gray, respectively. The SAR subclades corresponding to Musketeer proteins are shown in blue (ATHOS), purple (PORTHOS), dark blue (DARTAGNAN), and pink (ARAMIS). (**b**) Schematic representation of the functional domains of *Phytophthora palmivora* Musketeer proteins. IDR: intrinsically disordered region. (**c**) AlphaFold models of the DIX-like domains of *P. palmivora* Musketeer proteins, compared with the crystal structure of *Homo sapiens* DISHEVELLED 2 (HsDVL2) DIX domain (PDB entry: 4wip) and the AlphaFold model of *Arabidopsis thaliana* SOSEKI 1 (AtSOK1) DIX-like domain. Arrowheads indicate the L1 loop. For AlphaFold models, per-residue confidence (pLDDT) is color-coded as a gradient transitioning from magenta (low confidence) to cyan (high confidence). Scale bar: 1 nm.

We screened eukaryotic genomes for DDPs using reference DIX domain sequences from animals and plants (Fig. 1; Files S1 and 2). This analysis identified 1,452 protein candidates grouped into eleven subclades (Fig. 1a). Seven subclades included well-known animal and plant proteins (DVL, AXIN, DIXIN, and SOSEKI), while four were unique to the SAR supergroup. The latter included proteins from prominent plant and animal pathogens, such as *Phytophthora* spp. and malaria parasites. Notably, 97% of SAR species in our data set exhibit a motile stage in their lifecycle. Among exceptions are algae (e.g., *Nannochloropsis*) and some downy mildews (*Hyaloperonospora*, *Bremia*).

Next, we analyzed the functional domain composition of DDPs across the SAR supergroup (Fig. 1b; Fig. S1 and S2). All proteins comprised an N-terminal DIX-like domain, followed by an intrinsically disordered region of varying length containing short linear motifs (SLiMs) (Fig. 1b; Fig. S1 and S3). Proteins from three subclades also featured additional C-terminal domains (Fig. S2). To highlight their shared DIX domain and distinct C-terminal content, we named these DDPs after characters from the famous novel *The Three Musketeers* by Alexandre Dumas. PORTHOS proteins were named after their C-terminal protein kinase domain, while ARAMIS and DARTAGNAN proteins carried engulfment and cell motility (ELMO) and DnaJ domains, respectively. The proteins lacking a C-terminal domain were called ATHOS.

The DIX-like domains of Musketeer proteins exhibited a typical ubiquitin-like fold, with five β-strands and an α-helix (Fig. 1c; Fig. S4). In particular, ATHOS and DARTAGNAN DIX-like domains exhibited structural similarity to *Homo sapiens* DVL2 and *Arabidopsis thaliana* SOK1. In contrast, ARAMIS and PORTHOS DIX-like domains had larger L1 loops, indicating potential functional divergence (Fig. 1c; Fig. S4). A subset of proteins within the ATHOS subclade carried DIX-like domains with L1 loops similar to the DVL2 DIX domain (4), SLiMs reminiscent of those found in ATHOS proteins, and a kinase domain from the mitogen-activated protein (MAP) kinase subfamily, distinct from the NAK kinase domains found in PORTHOS proteins (Fig. S5 to S7). These proteins were found exclusively in oomycete pathogens from the genera *Bremia*, *Globisporangium*, *Pythium*, and *Phytophthora* (Fig. S7).

We further assessed structural homology by comparing Musketeer DIX-like models with the crystal structures of DIX domains from *Homo sapiens* DVL2, DIXIN (Ccd1), *Rattus norvegicus* AXIN1, and *Arabidopsis thaliana* SOK4. As a control, we included the *Homo sapiens* ELMO2 Ras-binding domain (RBD) due to its ubiquitin-like fold (Fig. S8). All DIX and DIX-like domains showed structural similarity scores (DALI Z-scores) above 8.5, suggesting structural similarity. In contrast, comparisons with the RBD domain produced consistently lower Z-scores (below 5.5), highlighting their structural divergence (Fig. S8). These findings reveal the presence of structurally plausible DIX-like domains within the SAR supergroup, forming novel DDPs that may reflect functional specialization in these organisms.

### *Phytophthora palmivora* ATHOS localizes in the ventral groove of zoospores

**Fig 2:**
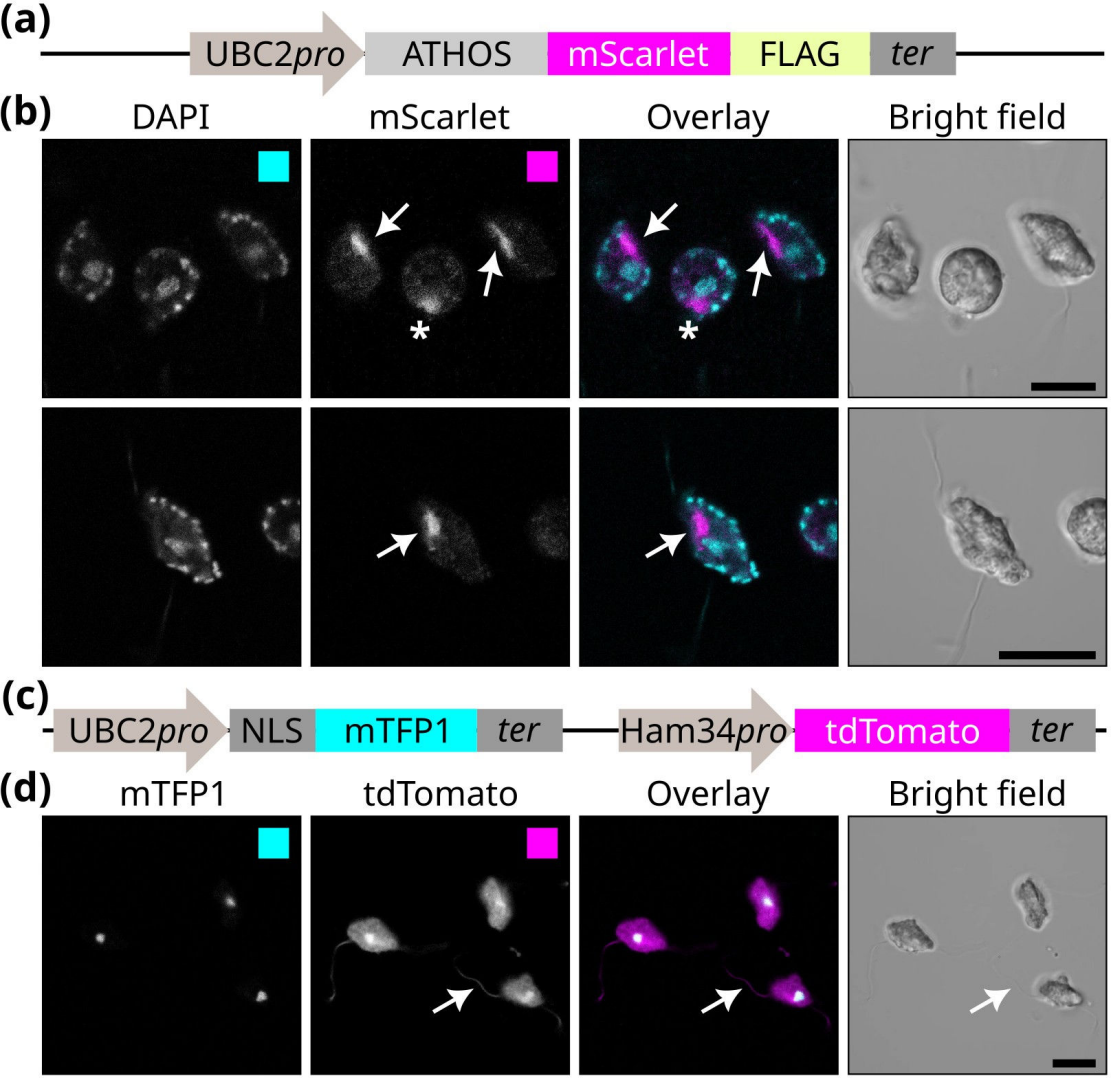
*P. palmivora* ATHOS accumulates near the ventral groove of zoospores. (**a**) Schematic representation of the reporter cassette used to assess the subcellular localization of *P. palmivora* ATHOS. (**b**) Representative images of immobilized *P. palmivora* zoospores showing the accumulation of ATHOS proteins in the ventral groove (arrows) and its persistence in newly formed cysts (asterisk). Nuclei and mitochondria were stained with DAPI. (**c**) Schematic representation of the dual reporter cassette used to label nuclei (NLS-mTFP1) and cytoplasm (tdTomato). (**d**) Representative images of immobilized *P. palmivora* zoospores co-expressing nuclear-localized mTFP1 and a cytoplasmic tdTomato. The arrow indicates tdTomato signal in flagella. Scale bars: 10 µm.

We further investigated the biological role of Musketeer proteins, focusing on ATHOS, by analyzing its localization in *P. palmivora* zoospores. *P. palmivora* was chosen as a model because all Musketeer proteins are transcriptionally expressed in this species (7). First, we confirmed that ATHOS transcripts accumulate at the zoospore stage (Fig. S9). We generated a transgenic *P. palmivora* line expressing ATHOS fused to mScarlet-FLAG, under the control of the *UBC2* promoter. Using confocal microscopy on immobilized living zoospores, we observed that ATHOS accumulates at a specific site near the ventral groove, close to the flagellar insertion point (Fig. 2a and b; Fig. S10). By contrast, zoospores from a transgenic line expressing a cytoplasmic tdTomato (8) show homogeneous red fluorescence within cell bodies and flagella (Fig. 2c and d). Furthermore, expression of an mCitrine-Centrin 2 fusion reporter (8) resulted in fluorescence accumulation in punctate structures, centrosomes, and flagella (Fig. S11). The accumulation of ATHOS proteins near the flagellar insertion point suggests that they may play a role in motility-related processes.

### The *P. palmivora* Musketeer proteins ATHOS and DARTAGNAN physically interact *in vivo*

**Fig 3:**
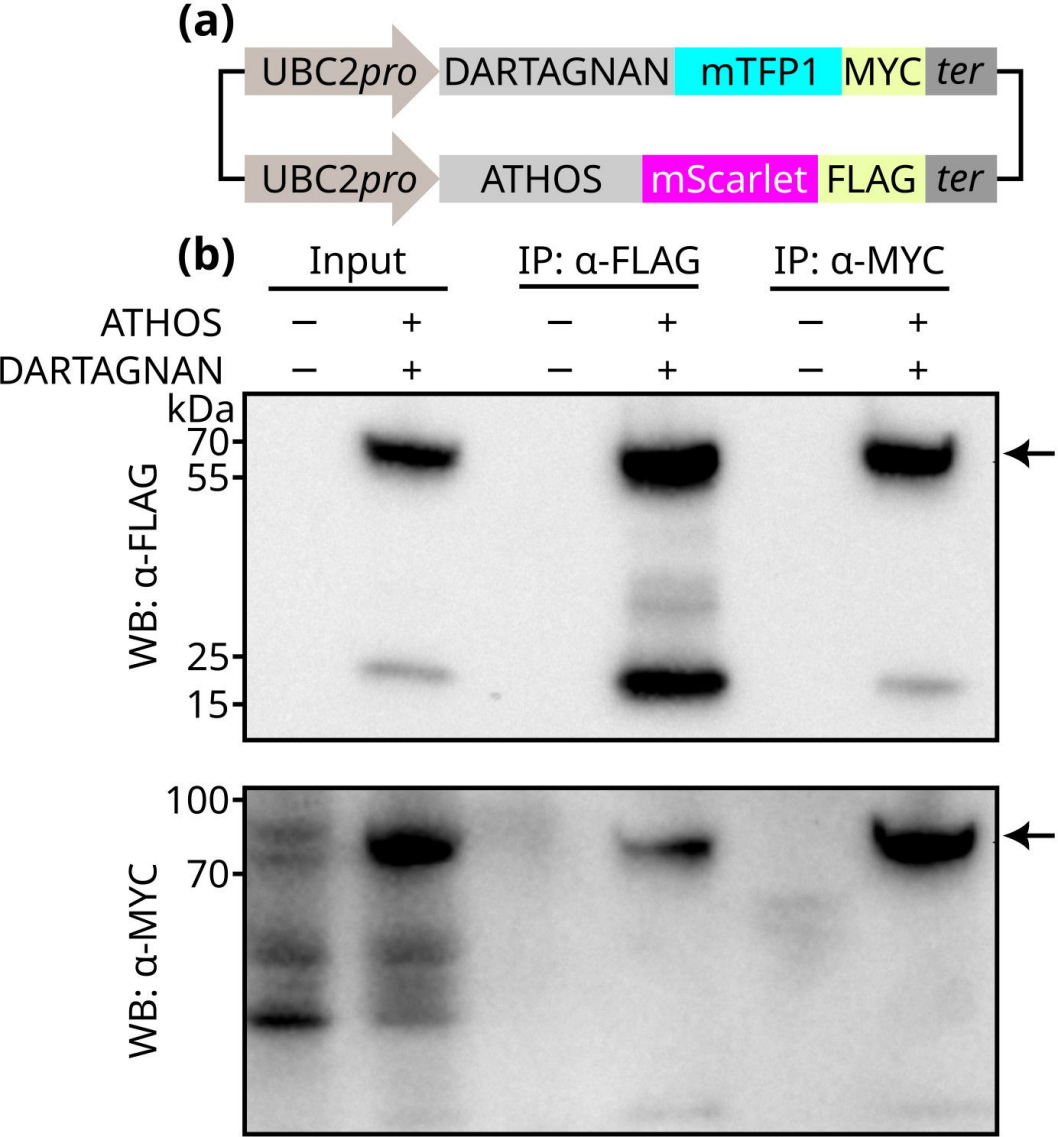
*Phytophthora palmivora* ATHOS and DARTAGNAN physically interact *in vivo*. (**a**) Schematic representation of the expression cassettes for *P. palmivora* ATHOS and DARTAGNAN. UBC2: *P. palmivora* ubiquitin-conjugating enzyme promoter. Transformants were selected on geneticin (G418). (**b**) MYC-tagged DARTAGNAN co-purifies with FLAG-tagged ATHOS upon FLAG pulldown, and *vice versa*. IP: immunoprecipitation; WB: Western blot. Arrows indicate the expected protein sizes.

We examined whether Musketeer proteins interact with each other *in vivo*. Using the transgenic *P. palmivora* line expressing ATHOS-mScarlet-FLAG described above, we performed FLAG pulldown assays followed by liquid chromatography–tandem mass spectrometry (LC-MS/MS). This analysis identified spectra corresponding to ARAMIS and DARTAGNAN, suggesting potential interactions between ATHOS and other Musketeer proteins (File S3). To validate these interactions, we co-expressed FLAG-tagged ATHOS with Myc-tagged DARTAGNAN or ARAMIS in *P. palmivora*. Of the combinations tested, we successfully generated strains co-expressing ATHOS and DARTAGNAN. FLAG pulldown from total protein extracts confirmed the co-purification of DARTAGNAN with ATHOS (Fig. 3). Reciprocally, Myc pulldown confirmed that ATHOS co-purified with DARTAGNAN (Fig. 3). Thus, at least three Musketeer proteins interact in *P. palmivora* mycelium, and ATHOS and DARTAGNAN may participate in similar molecular processes.

We identified several DDPs featuring novel domain combinations that have not been previously observed in eukaryotes. Notably, none of the DDPs characterized to date include ELMO, DnaJ, or kinase domains. Musketeer proteins are thus a clear example of the unique domain pairings (bigrams) commonly found in oomycetes (9). For instance, ELMO domains are known to occur in two protein classes: ELMOs and ELMODs. ELMOD proteins trace back to the last common ancestor of all eukaryotes, whereas ELMO proteins are specific to the Opisthokonta clade, which contains fungi and metazoans (10). ARAMIS proteins constitute a third class of ELMO domain-containing proteins, combining DIX-like and ELMO domains. Similarly, the co-occurrence of DIX-like domains with DnaJ in DARTAGNAN and NAK-like kinase domains in PORTHOS has not been observed in other DDPs. However, it is noteworthy that DnaJ and NAK kinase domains co-occur in the cyclin G-associated kinase (GAK) (11, 12). It is tempting to speculate that DARTAGNAN and PORTHOS proteins may reconstitute a GAK-type activity through interactions mediated by their DIX-like domains. Hence, our findings reveal previously uncharacterized domain architectures in DDPs and highlight their evolutionary plasticity. The aggregation properties of DIX-like domains may have been recruited in the SAR supergroup to perform functions handled by single proteins in other lineages.

Except for an unconfirmed sequence in the chytrid fungus *Batrachochytrium salamandrivorans,* which resembles ATHOS (KAH9255096.1), Musketeer proteins represent a unique innovation within the SAR supergroup. Notably, 97% of SAR species encoding Musketeer proteins are either micro-swimmers or have a motile stage in their life cycle. They include zoospore-forming oomycetes (13), ciliates (14), and apicomplexans, such as *Toxoplasma* and *Plasmodium*, which produce flagellated male gametes called microgametes (15). Exceptions include several downy mildews from the genera *Hyaloperonospora*, *Bremia,* and *Peronospora*, which also encode Musketeer proteins. Since a *Phytophthora* ATHOS protein localizes near the zoospore ventral groove, one can hypothesize that Musketeer proteins may be involved in motility-related processes. Consistent with this hypothesis, Musketeer proteins were not identified in the *Rhizaria* clade, which contains predominantly predatory amoeboid species (16). Other genes have been associated with motile stages in oomycetes. For instance, in the late blight pathogen *Phytophthora infestans*, the Cdc14 phosphatase is expressed exclusively in the spore stages of the life cycle and accumulates at basal bodies (17, 18). However, Cdc14 and other stage-specific motility genes, but not Musketeer proteins, are absent from the genomes of downy mildews that do not produce swimming zoospores, such as *Hyaloperonospora* and *Bremia* (19). Since the genes encoding Musketeer proteins are constitutively expressed (7), Musketeer proteins likely play a role at different life stages and are thus not specific to motility. Future research will investigate the molecular interactors of Musketeer proteins and their role at other life stages. For instance, oomycete Musketeer proteins may also be implicated in the development of infection structures, such as appressoria and haustoria.

### Conclusion

This study reveals the unexpected complexity of DDPs across eukaryotic lineages, with unique domain combinations in Musketeer proteins suggesting distinct functional roles compared with animal and plant DDPs. Our findings provide valuable insights into the DIX-like domains and functional diversification within the SAR supergroup. Future studies will focus on characterizing the functions of Musketeer during mycelium development and host interactions. Such investigations may reveal fundamental morphogenetic processes in the SAR supergroup and lay the groundwork for novel strategies to combat oomycete diseases in plants and animals by altering zoosporogenesis, zoospore swimming capabilities, mycelium development, or the formation of infectious structures.

### Microbial strains and growth conditions

*P. palmivora* Butler isolate LILI (accession no. P16830) was initially isolated from oil palm in Colombia and maintained in the *Phytophthora* collection at the Agrobiotech Institute (Sophia Antipolis, France). *P. palmivora* strains were maintained on Petri plates of V8 agar (1.5% agar) at 25°C. Transformed strains were maintained in the presence of geneticin (G418) at a final concentration of 100  mg/L. Mycelium was grown under constant light conditions. For zoospore production, 7-day-old plates were incubated at 4°C for 30  min. Plates were then flooded with sterile water and incubated at room temperature for 15  min.

### *In silico* analyses

DDP candidates were retrieved from public databases using protein BLAST and hidden Markov model searches. Amino acid sequences were aligned using the MAFFT alignment program (20) with default settings. After alignment, phylogenetic relationships were investigated using IQ-TREE (21). SLiMs were identified on amino acid sequence alignments and checked with the Eukaryotic Linear Motif (ELM) database (http://elm.eu.org/). Protein structures were modeled with AlphaFold 2 (22). Structural homology was assessed with the DALI web server (http://ekhidna2.biocenter.helsinki.fi/dali/). Large-scale modeling of Musketeer proteins was performed by Tamarind Bio (https://www.tamarind.bio/). Protein models were rendered with UCSF ChimeraX (https://www.cgl.ucsf.edu/chimerax/).

### Plasmid construction

*P. palmivora* ATHOS (PLTG_03873) and DARTAGNAN (PLTG_06708) (7) sequences were amplified from complementary DNA obtained from 2-day-old *P. palmivora* mycelium grown on liquid V8 medium. Sequences were fused to either mScarlet:FLAG or mTFP1:Myc reporters by overlap extension PCR. Amplicons were cloned into a modified pGEM-T vector containing a geneticin (G418) selection cassette derived from pTORKm34GW (8) using NEBuilder HiFi DNA Assembly (New England Biolabs).

### *P. palmivora* transformation

*P. palmivora* was transformed by electroporation. Mixes containing zoospores, 20 µg plasmid DNA, and modified Petri’s solution (final concentration: 0.25 mM CaCl_2_, 1 mM MgSO_4_, 1 mM KH_2_PO_4_, and 0.8 mM KCl) were electroporated using a Gene Pulser Xcell Electroporation System (Bio-Rad) with exponential decay using the following settings: voltage 500 V, capacitance 50 µF, resistance 800 ohm. After electroporation, zoospores were incubated for 8 h at 25°C in liquid V8 medium with gentle shaking. Transformants were selected on V8-agar plates containing 100 mg/L geneticin (G418).

### Confocal microscopy

For microscopic observations, zoospores were immobilized in 100 mM lithium chloride at 4°C (23) and immediately stained with 4′,6-diamidino-2-phenylindole (DAPI) (Thermo Fisher) or MitoTrack Orange CMTMRos (ABP Biosciences). Zoospores were observed using a Zeiss LSM 880 confocal laser scanning microscope equipped with a 63× NA 1.2 water immersion objective. Wavelengths of 405 and 561 nm were used for the excitation of DAPI and mScarlet, respectively. Microscopic pictures were analyzed with ImageJ (https://imagej.nih.gov/ij/).

### Protein extraction, immunoprecipitation, and immunoblot

Protein extraction, immunoprecipitation, and immunoblot were performed as previously described (24), except that total proteins were extracted from *P. palmivora* mycelium grown in a liquid V8 medium. For immunoblots, bands were revealed by chemiluminescence ECL (Thermo Fisher) using a Chemidoc MP Imaging System (Bio-Rad).

### Mass spectrometry data

After SDS-PAGE, two gel pieces spanning the entire lane were excised and used for in-gel reduction and alkylation, followed by digestion of the proteins with trypsin and peptide extraction. Peptides were analyzed by liquid chromatography with tandem mass spectrometry at the Cambridge Proteomics Centre (Cambridge, UK). Proteins were identified using a *P. palmivora* database derived from *de novo* transcriptome assembly (7).

## Data Availability

The mass spectrometry proteomics data have been deposited to the ProteomeXchange Consortium via the PRIDE (25) partner repository with the data set identifier PXD056467.

## SUPPLEMENTAL MATERIAL

File S1 mbio.03966-24-s0001.xlsx DDP table.

File S2 mbio.03966-24-s0002.txt DIX hidden Markov model.

File S3 mbio.03966-24-s0003.xlsx ATHOS mass spectrometry data.

Supplemental figures mbio.03966-24-s0004.pdf Figures S1 to S11.

ASM does not own the copyrights to Supplemental Material that may be linked to, or accessed through, an article. The authors have granted ASM a non-exclusive, world-wide license to publish the Supplemental Material files. Please contact the corresponding author directly for reuse.
