# Supplementary material for: Diversification of DIX domain-containing proteins in the SAR supergroup
Source: mBio. 2025 Apr 16;16(5):e03966-24. doi: 10.1128/mbio.03966-24 (PMC12077222; doi:10.1128/mbio.03966-24)
Supplement: Supplemental figures — Figures S1 to S11. [file mbio.03966-24-s0004.pdf]

Supporting Information

Diversification of DIX domain-containing proteins in the SAR supergroup

Maria-Myrto Kostareli, Timo Westerink, Gabriel Couillaud, Maaria Peippo, Francine Govers, Dolf Weijers, Edouard Evangelisti

Supporting Files

Supporting File 1: Table of DIX and DIX-like domain-containing proteins used in this study.

Supporting File 2: Hidden Markov Model (HMM) used to identify DIX-like domains.

Supporting File 3: Protein spectra identified upon *Phytophthora palmivora* ATHOS pulldown.

Supporting Figures

Figure S1. Intrinsically disordered regions separate DIX-like and C-terminal domains in *Phytophthora palmivora* Musketeer proteins PORTHOS, ARAMIS, and DARTAGNAN 2

Figure S2. AlphaFold models of the C-terminal domains of PORTHOS, ARAMIS, and DARTAGNAN. 3

Figure S3. The intrinsically disordered regions of ATHOS and DARTAGNAN contain conserved short linear motifs (SLiMs) 4

Figure S4. Structural overlay of *Phytophthora palmivora* DIX-like domains and similar domains occurring in the SAR supergroup 5

Figure S5. Phylogenetic relationships of PORTHOS kinase domains with the kinome of *Phytophthora infestans*. 6

Figure S6. AlphaFold model of the kinase domain of *Bremia lactucae* PORTHOS-like protein 7

Figure S7. Several oomycete PORTHOS-like proteins carry a DIX domain and short linear motifs reminiscent of ATHOS proteins 8

Figure S8. *Phytophthora palmivora* DIX-like domains are structurally similar to animal and plant DIX domains 9

Figure S9. Transcripts encoding the ATHOS protein accumulate in *Phytophthora palmivora* zoospores 10

Figure S10. Subcellular localization of *Phytophthora palmivora* ATHOS in zoospores and early cysts 11

Figure S11. Subcellular localization of *Phytophthora palmivora* Centrin 2 in zoospores 12

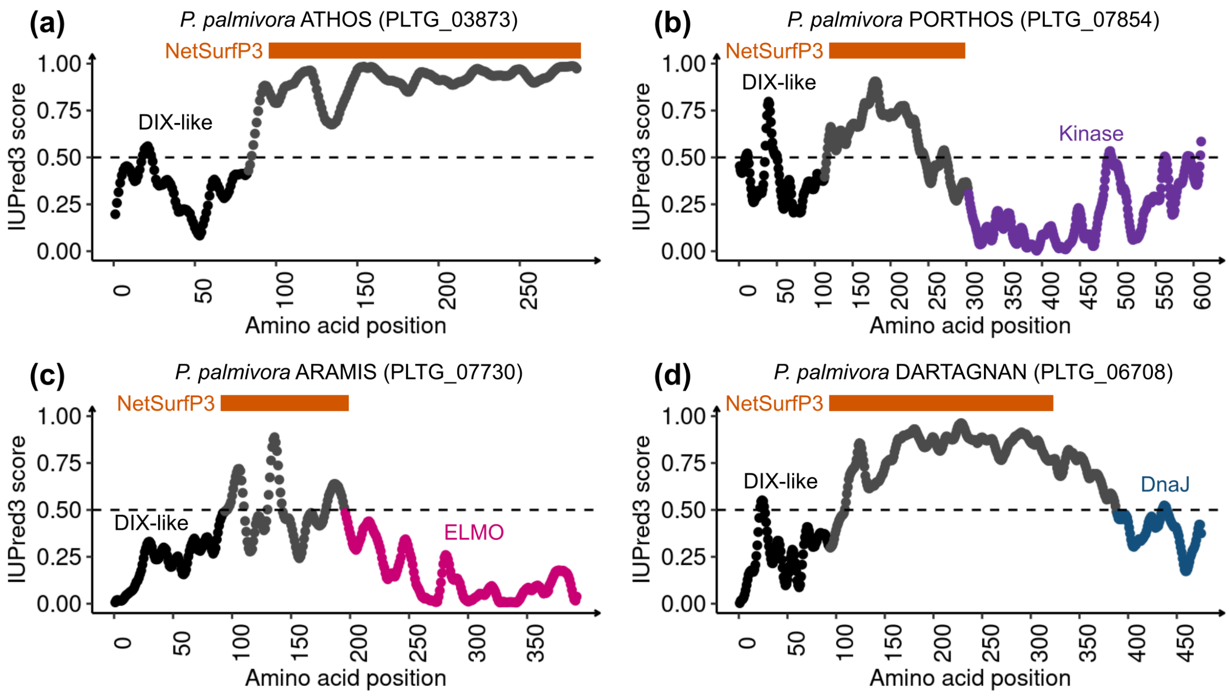

Figure S1. Intrinsically disordered regions separate DIX-like and C-terminal domains in *Phytophthora palmivora* Musketeer proteins PORTHOS, ARAMIS, and DARTAGNAN. (a-d) The amino acid sequences of *P. palmivora* Musketeer proteins ATHOS (PLTG_03873) **(a)**, PORTHOS (PLTG_07854) **(b)**, ARAMIS (PLTG_07730) **(c)**, and DARTAGNAN (PLTG_06708) **(d)** were analyzed for intrinsically disordered regions using UIPred3 (Erdős *et al.*, 2021) and NetSurfP3 (Høie *et al.*, 2022). The graphs display UIPred3 scores along the sequences. DIX-like domains are highlighted in black, while C-terminal domains are indicated in purple (kinase), pink (ELMO), or blue (DnaJ). Qualitative predictions from NetSurfP3 are shown in orange.

References:

Erdős G, Pajkos M, Dosztányi Z (2021). IUPred3: prediction of protein disorder enhanced with unambiguous experimental annotation and visualization of evolutionary conservation. *Nucleic Acids Research* 49 (W1): W297–W303.

Høie MH, Kiehl EN, Petersen B, Nielsen M, Winther O, Nielsen H, Hallgren J, Marcatili P (2022). NetSurfP-3.0: accurate and fast prediction of protein structural features by protein language models and deep learning. *Nucleic Acids Research* 50 (W1): W510–W515.

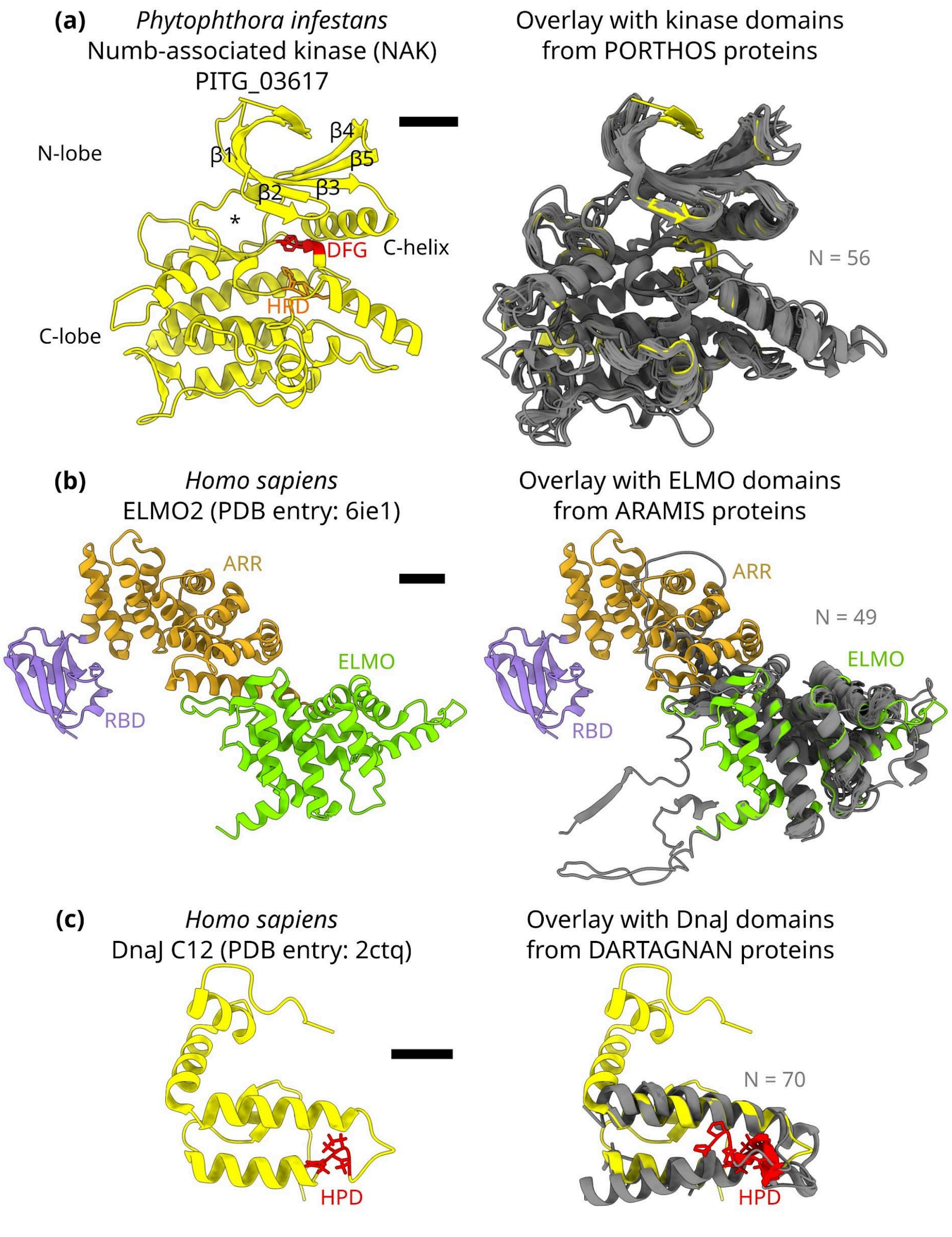

Figure S2. AlphaFold models of the C-terminal domains of PORTHOS, ARAMIS, and DARTAGNAN. (a) Numb-associated kinase (NAK)-like kinase domains of PORTHOS proteins (grey), overlaid with the AlphaFold model of *Phytophthora infestans* NAK (PITG_03617) (yellow). The conserved DFG and HRD motifs are highlighted in red and yellow, respectively. Asterisks indicate the ATP binding pocket. **(b)** Engulfment and cell motility (ELMO) domains of ARAMIS proteins (grey) compared to the crystal structure of *Homo sapiens* ELMO2 (PDB entry: 6ie1). The Ras-binding domain (RBD), armadillo repeats domain (ARR), and ELMO domain are shown in purple, orange, and green, respectively. **(c)** J domains of DARTAGNAN proteins (grey) overlaid with the J domain of *Homo sapiens* DnaJ C12 (PDB entry: 2ctq) (yellow). The DnaK-binding sequence HPD is shown in red. N indicates the number of overlaid domains. Scale bars: 1 nm.

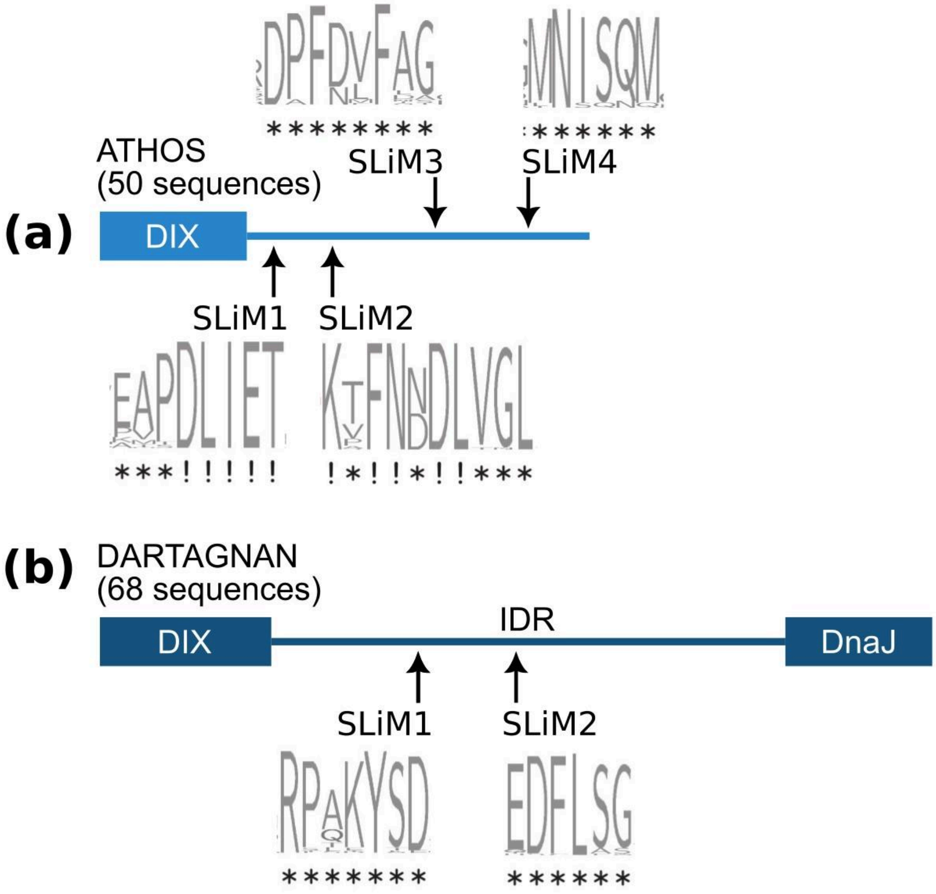

Figure S3. The intrinsically disordered regions of ATHOS and DARTAGNAN contain conserved short linear motifs (SLiMs). (a) ATHOS SLiMs were identified from a subset of 50 sequences from oomycete species. **(b)** DARTAGNAN SLiMs were identified from a subset of 68 sequences. In the sequence logos, letter size reflects the sequence conservation level.

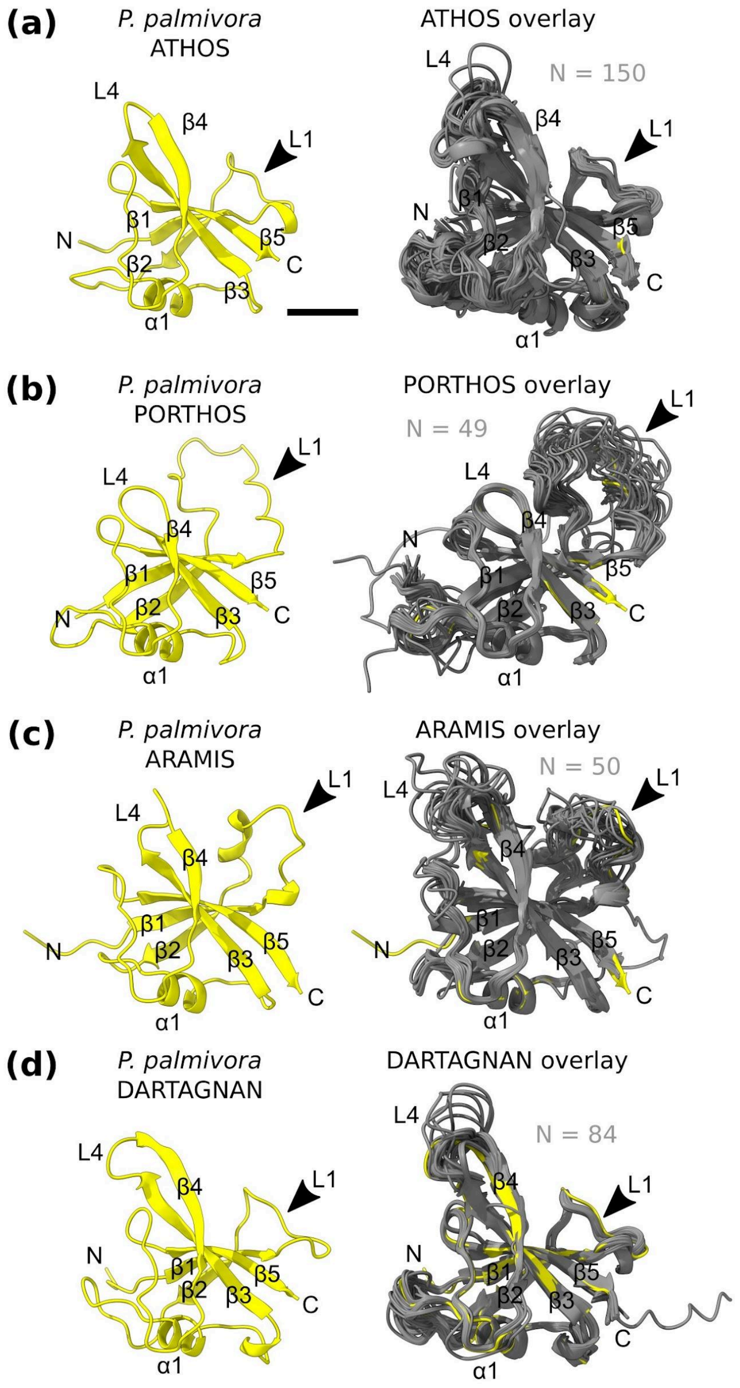

Figure S4. Structural overlay of *Phytophthora palmivora* DIX-like domains and similar domains occurring in the SAR supergroup. (a-d) Alphafold models of DIX-like domains from the *P. palmivora* Musketeer proteins ATHOS **(a)**, PORTHOS **(b)**, ARAMIS **(c)**, and DARTAGNAN **(d)** (yellow) were overlaid with DIX-like domains from similar proteins within the SAR supergroup (grey). N indicates the number of overlaid domains. Scale bar: 1 nm.

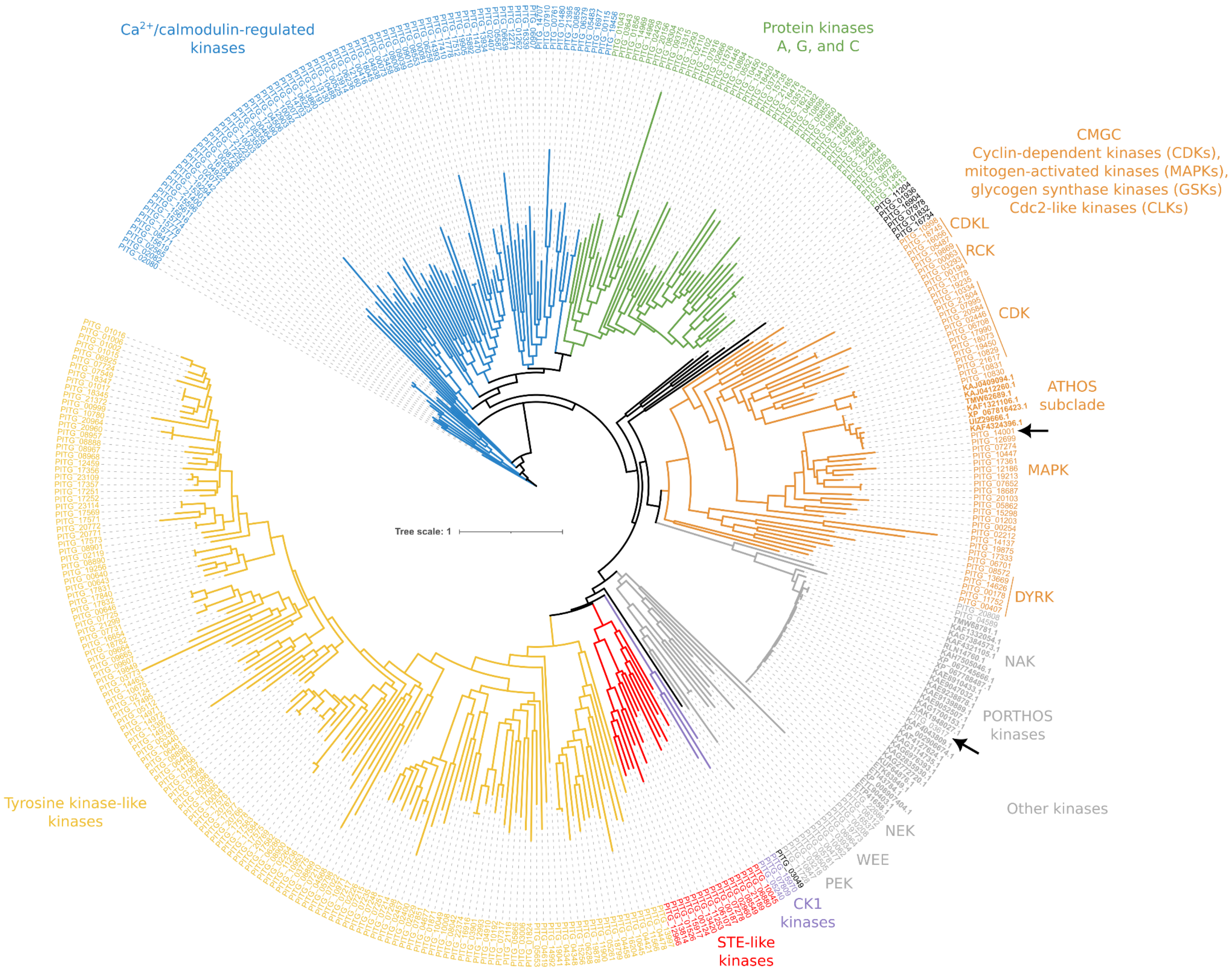

Figure S5. Phylogenetic relationships of PORTHOS kinase domains with the kinome of *Phytophthora infestans*. Illustrated is a maximum likelihood tree based on catalytic domains. Groups are highlighted and color-coded according to Judelson & Ah-Fong (2010). Arrows indicate the *P. infestans* kinases with the highest amino acid sequence similarity to PORTHOS kinases.

Reference:

Judelson HS, Ah-Fong AM (2010). The kinome of *Phytophthora infestans* reveals oomycete-specific innovations and links to other taxonomic groups. *BMC Genomics* 11: 700.

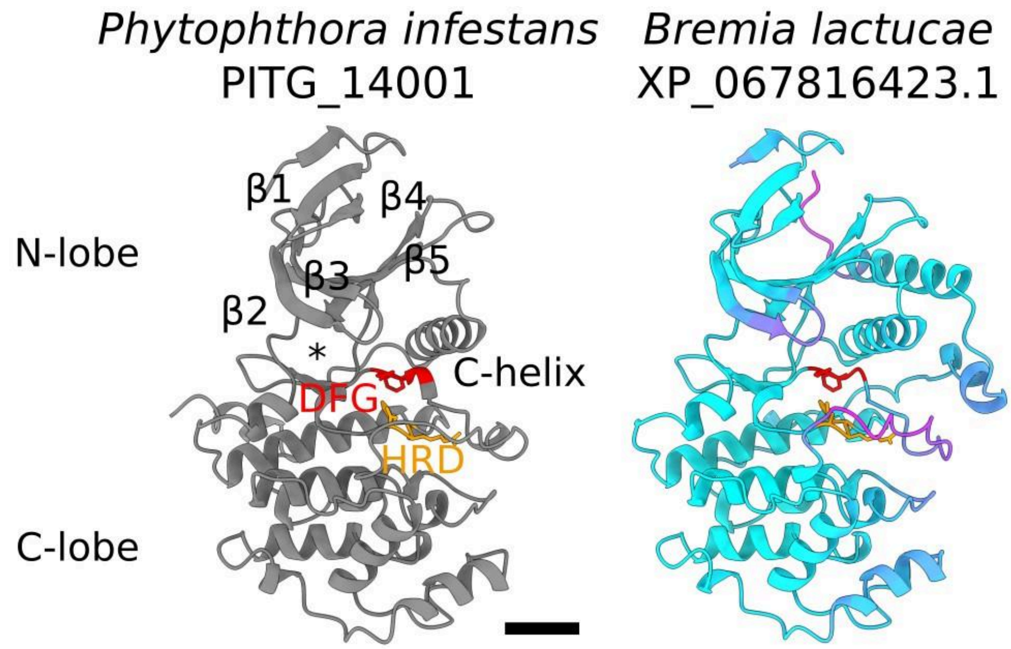

Figure S6. AlphaFold model of the kinase domain of *Bremia lactucae* PORTHOS-like protein. *B. lactucae* PORTHOS-like protein features a mitogen-activated protein kinase (MAPK)-type kinase domain with a functional catalytic site. The conserved DFG and HRD motifs are highlighted in red and yellow, respectively, while asterisks indicate the ATP binding pocket. Model confidence is represented by a color gradient transitioning from magenta (low confidence) to cyan (high confidence). Scale bar: 1 nm.

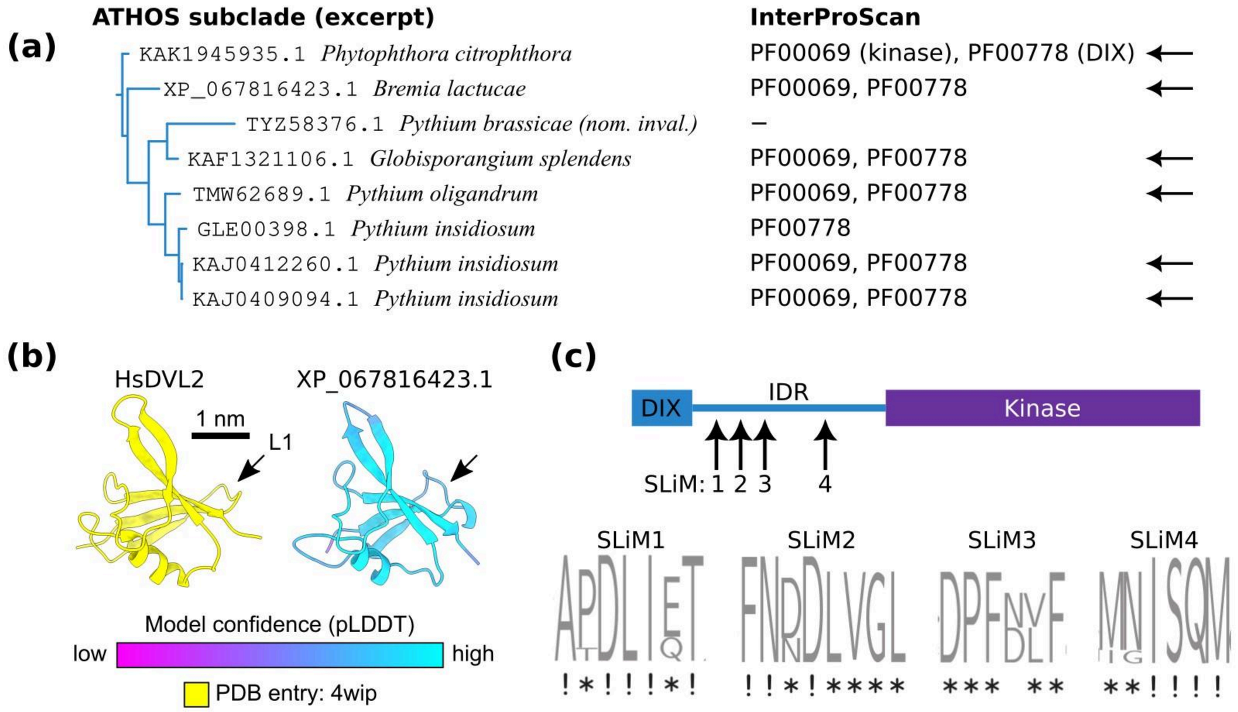

Figure S7. Several oomycete PORTHOS-like proteins carry a DIX domain and short linear motifs reminiscent of ATHOS proteins. (a) Detailed view of an ATHOS subclade containing PORTHOS-like proteins that possess DIX (PF00778) and protein kinase (PF00069) domains, indicated by arrows. **(b)** AlphaFold model of the DIX domain of *Bremia lactucae* protein XP_067816423.1. The crystal structure of *Homo sapiens* DISHEVELLED 2 (HsDVL2) DIX domain was used as a reference. Scale bar: 1 nm. **(c)** Short linear motifs (SLiMs) identified in the intrinsically disordered regions of PORTHOS-like proteins.

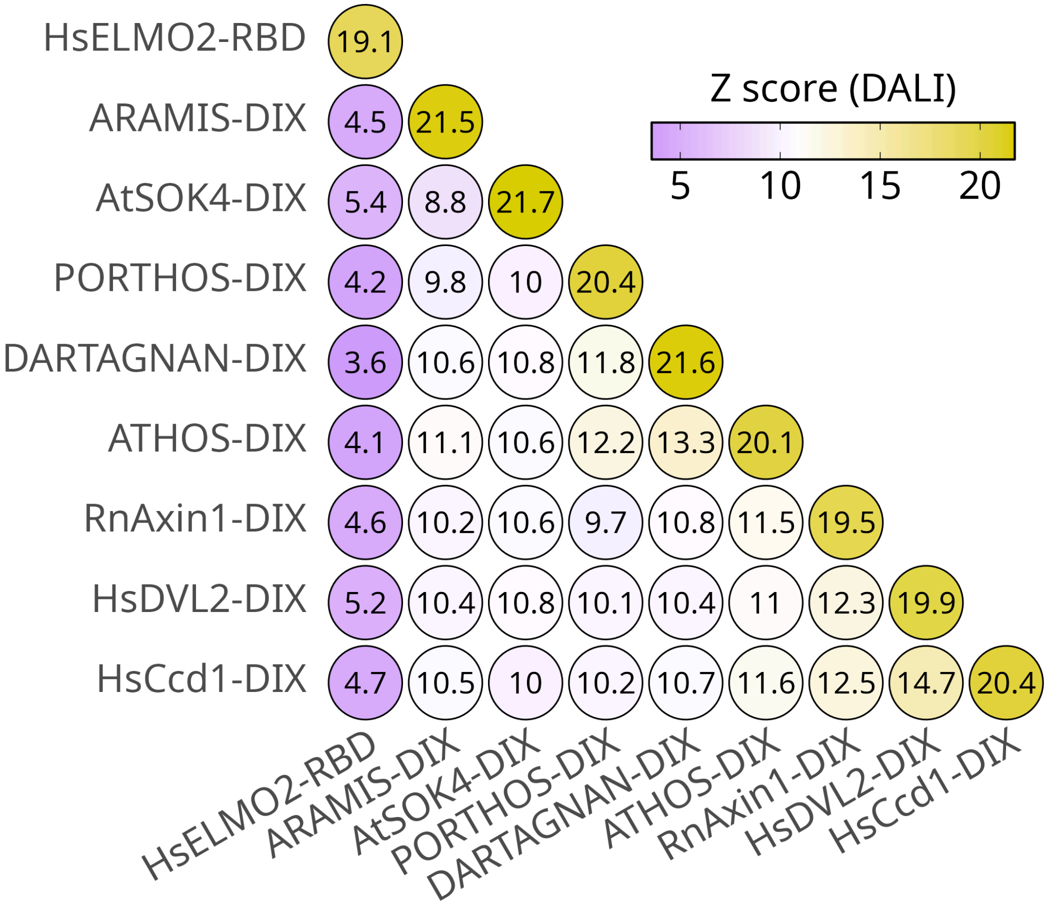

Figure S8. *Phytophthora palmivora* DIX-like domains are structurally similar to animal and plant DIX domains. AlphaFold models of DIX-like domains from the *P. palmivora* Musketeer proteins ATHOS, PORTHOS, ARAMIS, and DARTAGNAN were compared to those of *Homo sapiens* DVL2 (HsDVL2), DIXIN (HsCcd1), *Rattus norvegicus* Axin 1 (RnAxin1), and *Arabidopsis thaliana* SOK4 (AtSOK4) DIX domains using the DALI protein structure comparison server (Holm *et al.*, 2023). The Ras-binding domain (RBD) from *Homo sapiens* ELMO2 (HsELMO2) served as a control due to its ubiquitin-like fold. The numbers in the circles correspond to Z-scores, visually represented with a color gradient transitioning from purple (Z-score < 5) to yellow (Z-score > 15).

Reference:

Holm L, Laiho A, Törönen P, Salgado M (2023). DALI shines a light on remote homologs: One hundred discoveries. Protein Science 32(1):e4519.

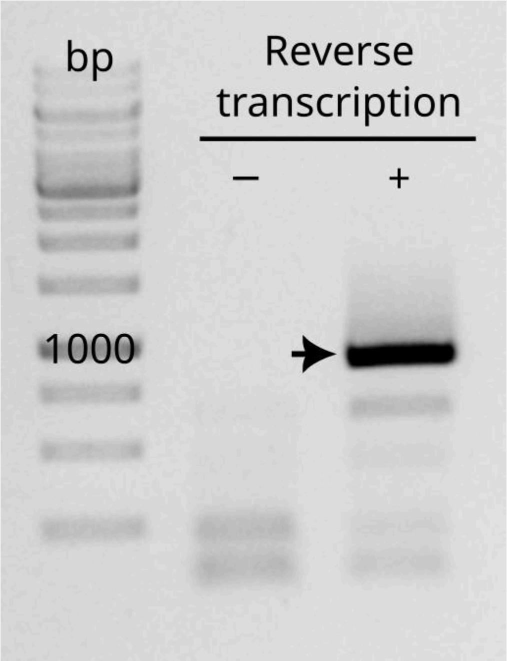

Figure S9. Transcripts encoding the ATHOS protein accumulate in *Phytophthora palmivora* zoospores. Total RNAs were extracted from *P. palmivora* zoospores and treated with DNase to remove DNA contamination. Purified RNAs were then used for reverse transcription using a blend of oligo(dT) and random hexamers (iScript cDNA Synthesis Kit, Bio-Rad). A reaction lacking reverse transcriptase (lane −) was used as negative control for RT-PCR. ATHOS was amplified using the forward primer ATHOS_F (5'-ATGGAGGGAATGCTCAACTACTACGTGC -3') and the reverse primer ATHOS_R (5'-CCATTGTCTCTGATTCGGGTTCCTCT-3'). The amplicon (arrow) was validated by Sanger sequencing (Eurofins Genomics, Germany). bp: base pairs.

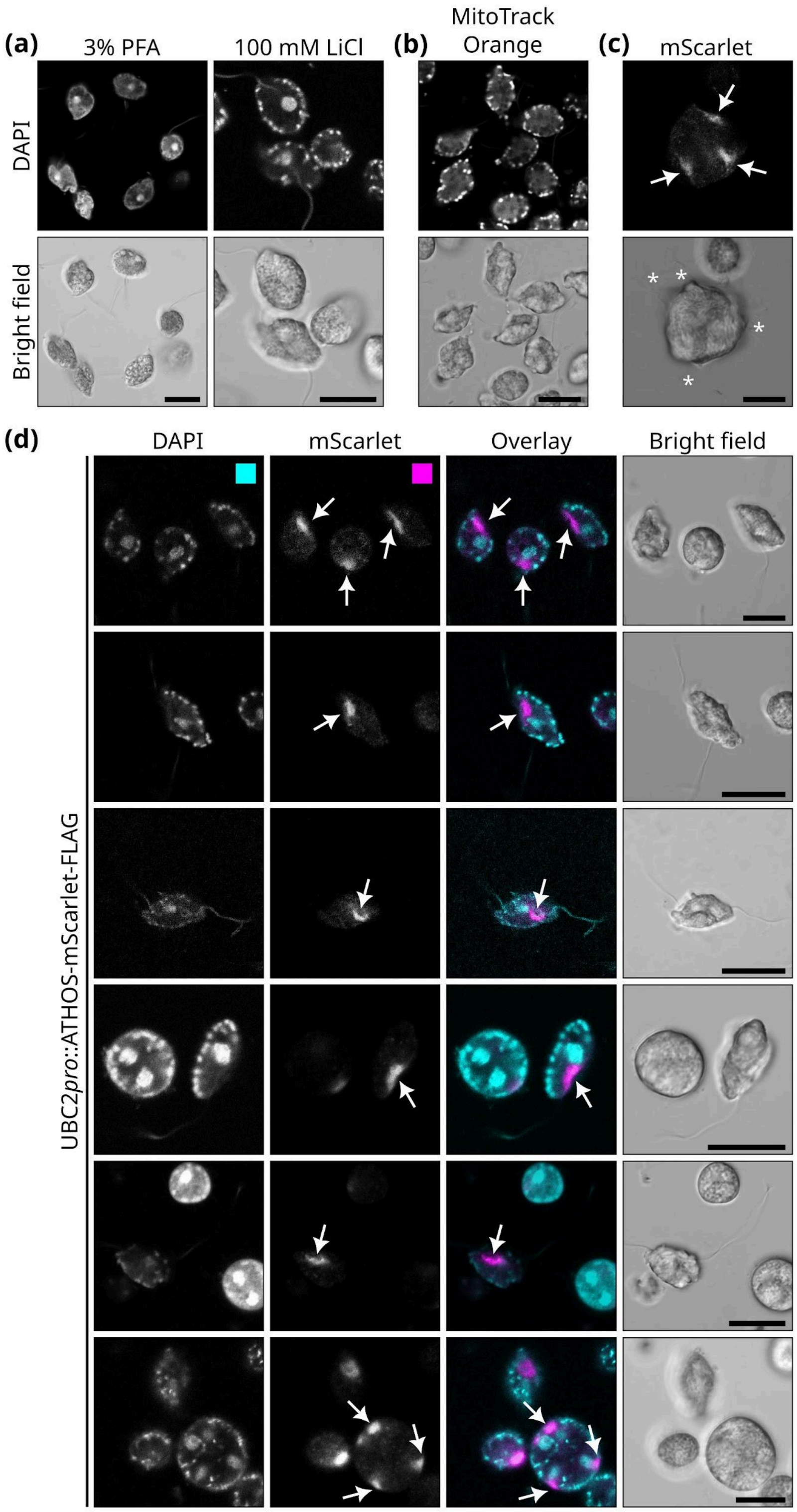

Figure S10. Subcellular localization of *Phytophthora palmivora* ATHOS in zoospores and early cysts. **(a)** Representative images of *P. palmivora* zoospores stained with DAPI after fixation with 3% paraformaldehyde (PFA, left) or non-lethal immobilization in the presence of 100 mM lithium chloride at 4°C (LiCl, right). **(b)** Representative images of *P. palmivora* zoospores stained with MitoTrack Orange CMTMRos after non-lethal immobilization in lithium chloride. **(c)** Abnormally shaped zoospore from a transgenic *P. palmivora* strain expressing the chimeric protein ATHOS-mScarlet under the control of the strong *UBC2* promoter. White arrows indicate ATHOS accumulations and white asterisks indicate flagella. **(d)** Subcellular localization of ATHOS in zoospores and early cysts. Zoospores were treated with lithium chloride and stained with DAPI before imaging. Arrows indicate ATHOS accumulations. Scale bars: 10 µm.

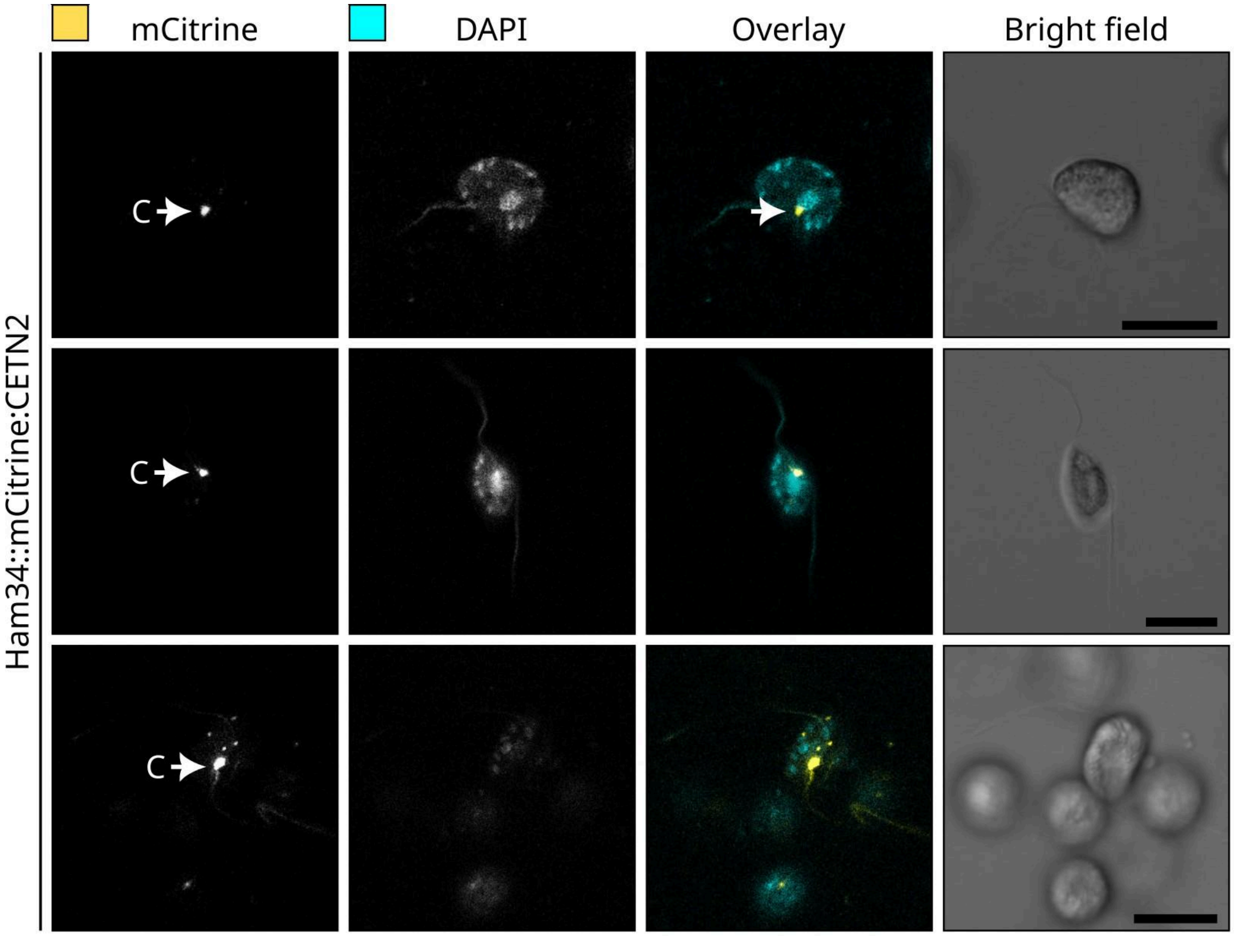

Figure S11. Subcellular localization of *Phytophthora palmivora* Centrin 2 in zoospores. Representative images of zoospores from a transgenic *P. palmivora* strain expressing a mCitrine-Centrin 2 fusion under the control of *Bremia lactucae* Ham34 promoter (Ham34::mCitrine-CETN2). Zoospores were treated with lithium chloride and stained with DAPI before imaging. C: centrosome. Scale bars: 10 µm.
